# H_2_-saturation of high affinity H_2_-oxidizing bacteria alters the ecological niche of soil microorganisms unevenly among taxonomic groups

**DOI:** 10.7717/peerj.1782

**Published:** 2016-03-10

**Authors:** Sarah Piché-Choquette, Julien Tremblay, Susannah G. Tringe, Philippe Constant

**Affiliations:** 1 INRS-Institut Armand-Frappier, Laval, Quebec, Canada; 2 Biomonitoring, National Research Council Canada, Montreal, Quebec, Canada; 3 DOE Joint Genome Institute, Walnut Creek, California, United States of America

**Keywords:** Soil, Microbial ecology, Correlation network

## Abstract

Soil microbial communities are continuously exposed to H_2_ diffusing into the soil from the atmosphere. N_2_-fixing nodules represent a peculiar microniche in soil where H_2_ can reach concentrations up to 20,000 fold higher than in the global atmosphere (0.530 ppmv). In this study, we investigated the impact of H_2_ exposure on soil bacterial community structure using dynamic microcosm chambers simulating soil H_2_ exposure from the atmosphere and N_2_-fixing nodules. Biphasic kinetic parameters governing H_2_ oxidation activity in soil changed drastically upon elevated H_2_ exposure, corresponding to a slight but significant decay of high affinity H_2_-oxidizing bacteria population, accompanied by an enrichment or activation of microorganisms displaying low-affinity for H_2_. In contrast to previous studies that unveiled limited response by a few species, the relative abundance of 958 bacterial ribotypes distributed among various taxonomic groups, rather than a few distinct taxa, was influenced by H_2_ exposure. Furthermore, correlation networks showed important alterations of ribotype covariation in response to H_2_ exposure, suggesting that H_2_ affects microbe-microbe interactions in soil. Taken together, our results demonstrate that H_2_-rich environments exert a direct influence on soil H_2_-oxidizing bacteria in addition to indirect effects on other members of the bacterial communities.

## Introduction

Soil microbial communities are continuously exposed to molecular hydrogen (H_2_). Trace levels of H_2_ (0.530 ppmv) diffuse into the soil from the global atmosphere, yet higher concentrations can be found in the rhizosphere of N_2_-fixing legumes (Constant, Poissant & Villemur, 2009). Indeed, H_2_ is an obligate by-product of nitrogenase in N_2_-fixing free living or symbiotic bacteria, with an H_2_ molecule produced for every reduced N_2_ molecule (Hoffman, Dean & Seefeldt, 2009). It has been estimated that 240,000 L of H_2_ are produced per hectare of legume crop over a growing season (Dong et al., 2003). At the microscale level, there is a steep H_2_ concentration gradient starting at about 20,000 ppmv at the soil-nodule interface and ending with sub-atmospheric levels a few centimeters away (La Favre & Focht, 1983; Rasche & Arp, 1989; Witty, 1991). Despite the high concentrations of H_2_ found in legumes rhizosphere, a very small proportion escapes to the atmosphere due to H_2_-oxidizing bacteria (HOB) thriving in soil. These microorganisms also play a vital role in the global budget of H_2_, being responsible for about 80% (60 Tg H_2_ yr^−1^) of the global losses of this trace gas from the atmosphere (Constant, Poissant & Villemur, 2009; Ehhalt & Rohrer, 2009).

Aerobic soil bacteria scavenging H_2_ diffusing from the atmosphere and N_2_-fixing nodules encompass a broad range of taxonomic groups. These microorganisms generally possess one or up to four different types of [NiFe]-hydrogenases catalyzing the interconversion of H_2_ into protons and electrons (H_2_↔2H^+^ + 2e^−^). These hydrogenases are classified into four distinct phylogenetic groups, each displaying particular physiological roles (Constant & Hallenbeck, 2012; Vignais & Billoud, 2007). Hydrogenases encompassing group 1h are generally characterized by a high affinity towards H_2_( _(app)_K_*m*_ < 100ppmv), conferring the ability to oxidize atmospheric H_2_ (Constant et al., 2010). Genome database mining of *hhyL* gene encoding the large subunit of high affinity hydrogenases are differentially distributed in *Actinobacteria*, with few representatives of *Proteobacteria*, *Acidobacteria* and *Chloroflexi* (Constant et al., 2011a; Constant et al., 2011b). The energy yield associated with the oxidation of atmospheric H_2_ is insufficient to support chemolithotrophic growth and compelling experimental evidence suggest that H_2_ supplies maintenance energy requirements and mixotrophic growth in high affinity HOB (Constant et al., 2011a; Constant et al., 2011b; Constant et al., 2010; Greening et al., 2014a; Greening et al., 2015). The distribution of the other [NiFe]-hydrogenase groups is broader, including methanogenic archaea and *Cyanobacteria* (Vignais & Billoud, 2007). *Ralstonia eutropha* and *Bradyrhizobium japonicum*, part of the knallgas bacteria functional group, are able to scavenge H_2_ diffusing from nodules in the presence of O_2_, yet they are unable to use atmospheric H_2_ due to the low-affinity ( _(app)_K_*m*_ > 1,000ppmv) and high H_2_ threshold concentration of their [NiFe]-hydrogenases (Conrad, Aragno & Seiler, 1983). Knallgas bacteria can use H_2_ as a sole or supplementary energy source in chemolithotrophic or mixotrophic growth, respectively. The co-occurrence of these two sub-populations of HOB, as defined by substrate affinity, is supported by the biphasic kinetics governing H_2_ oxidation activity in soil (Häring & Conrad, 1994).

Laboratory incubations simulating H_2_ fluxes from N_2_-fixing nodules demonstrated that soil bacterial community composition changed upon H_2_ exposure (Osborne, Peoples & Janssen, 2010; Stein et al., 2005; Zhang, He & Dong, 2009). However, methods previously used provided a low taxonomic resolution and coverage of bacterial communities responding to H_2_ exposure. Here, we revisited these experiments using a combination of high-throughput sequencing of the bacterial 16S rRNA gene and an H_2_ metabolism analysis to compare microbial community structure in dynamic microcosm chambers simulating soil H_2_ exposure from the atmosphere and from N_2_-fixing nodules, corresponding to unsaturating and saturating H_2_ concentrations for high affinity hydrogenases, respectively. Elevated H_2_ exposure was expected to activate and enrich HOB, consequently leading to indirect impacts on the whole soil bacterial community through competitive and synergistic microbe-microbe interactions.

## Materials and Methods

### Soil sample

Soil sample was collected in an agricultural land located in St. Claude (Québec, Canada) on the south shore of the St. Lawrence River (45.6809°N, −71.9969°W). The field is managed with fallow, potatoes and maize crop rotation. Potato seedlings (approximately 10 cm height) were present on the site during sampling in July 2013. The top layer of the A-horizon (0–10 cm depth) was collected, stored in plastic bags at 4 °C and processed within a week. Soil was air-dried for 48 h in the laboratory and sieved (2 mm mesh size) through a vibratory sieve shaker AS 200^®^ (Retsch GmbH, Haan, Germany) before preparation of soil microcosms. Soil was classified as sandy clay loam according to soil textural class parameters identified with the hydrometer method (Bouyoucos, 1936). Soil pH was determined with 1:2 soil-water suspensions with an Accumet pH-meter (Fisher Scientific, Hampton, NH). Total carbon (3.1 ± 0.3%) and total nitrogen (0.3 ± 0.0%) content were determined using an elemental combustion system using the protocol described in Khdhiri et al. (2015).

### Controlled H_2_ exposure in dynamic soil microcosm chambers

Dynamic microcosm chambers were designed to expose soil to controlled levels of H_2_ (Fig. S1). Microcosm chambers consisted of 0.9 L cell culture flasks (Corning, Tewksbury, MA, USA) equipped with a rubber stopper fitted with two 1/8” outside diameter PTFE (Teflon) tubes: the first supplied gas mixture to the microcosm chamber and the second was vented to the atmosphere. Synthetic gas mixtures supplied the microcosm chambers at a flowrate of 40 ml min^−1^, resulting in a dynamic headspace with a residence time of approximately 22 min in the enclosures. Gas mixtures were bubbled in water before entering in microcosms to prevent soil dryness. Two different H_2_ treatments were applied in parallel incubations. The first treatment (designated microcosms eH_2_(a) and eH_2_(b)), named elevated H_2_ (eH_2_), consisted of exposing soil to a dynamic headspace comprising 525 ppmv H_2_ in synthetic air, simulating H_2_ concentrations detected around N_2_-fixing nodules in soil (Hunt & Layzell, 1993). The second was a control treatment (designated microcosms aH_2_(a) and aH_2_(b)), named atmospheric H_2_ (aH_2_), where soil was exposed to a dynamic headspace comprising 0.54 ppmv H_2_ in synthetic air, representing H_2_ concentrations found in the atmosphere. Both treatments were replicated, resulting in four soil microcosms in total. Each microcosm chamber contained 200 g_(dw)_ soil at the beginning of the incubation period. This amount of soil ensured sufficient material for the monitoring of microbiological and physicochemical variables throughout the incubation period, while avoiding diffusion limitation of the H_2_ soil uptake rate measurements. Diffusion limitation was precluded since preliminary experiments using the same microcosm setup showed proportional H_2_ uptake activity as a function of the amount of soil in the chamber using 150, 200 and 250 g_(dw)_ soil samples (raw data file). Soil water content was adjusted to 20% water holding capacity before incubation for 10 days at 28 °C in the dark. Synthetic gas mixture supply was continuously maintained, with the exception of routine soil subsamples collection and high affinity H_2_ oxidation rate measurements (see below). A decrease in soil pH was observed over the course of the incubation (from pH 5.9 ± 0.2 to 5.1 ± 0.1) in all microcosms, without distinction between aH_2_ and eH_2_ treatments. Soil moisture was monitored using standard gravimetric method and maintained at 26 ± 5% throughout the incubation period. Blank (empty) microcosms incubated prior to the experiment did not show any H_2_ oxidation or production activity.

### H_2_ uptake activity

High affinity H_2_ oxidation activity was routinely monitored throughout the incubation of the soil microcosms. Briefly, microcosms were disconnected from their respective gas supply, flushed 5 min with a synthetic gas mixture (0.54 ppmv H_2_) and tightly closed with rubber septum caps. The 5-min flush was shown sufficient to avoid residual H_2_ degassing that would otherwise have led to an underestimation of H_2_ oxidation rate. A defined volume of H_2_ gas mixture (525 ± 10 ppmv H_2_ GST-Welco, PA, USA.) was injected to obtain an initial concentration of approximately 3 ppmv in the static headspace. Decrease of the H_2_ mixing ratio was monitored as a function of time by analyzing aliquots (10 ml) of the headspace air in a ta3000R gas chromatograph equipped with a reduction gas detector (Ametek Process Instruments^®^, DE, USA.) as previously described (Khdhiri et al., 2015). Considering the low level of H_2_ added in the headspace, H_2_ uptake reflected the activity of high affinity HOB, knallgas bacteria showing low affinity for H_2_ being unable to use these trace amounts of H_2_ (Conrad, Aragno & Seiler, 1983). The biphasic kinetic parameters governing H_2_ oxidation activity in soil (i.e., _(app)_K_*m*_ and _(app)_*V*_max_) were measured at the end of the 10-day incubation period after the addition of specified amounts of pure H_2_ into the headspace of the microcosms, as previously described (Schuler & Conrad, 1990).

### DNA extraction, qPCR and high-throughput sequencing of PCR-amplified bacterial 16S rRNA gene

Soil subsamples (approximately 10 g per subsample, without replacement) were collected in the four microcosms after 0, 1, 3, 5, 7 and 10 incubation days to investigate the taxonomic structure of microbial communities. The extraction of total genomic DNA was performed using the FastDNA SPIN kit for Soil^®^ (MP Biomedicals, Solon, OH, USA.). DNA was eluted in 100 µL nuclease-free water. Quality of the DNA was then examined on agarose gels and samples were quantified using Quantifluor dsDNA System^®^ (Promega, Fitchburg, WI, USA). The V4 region of bacterial 16S rRNA gene was PCR amplified (Table S1) and sequenced on an Illumina MiSeq 2000 instrument as multiplexed libraries to generate paired-end reads (2 × 250 bp). Quantification of bacterial 16S rRNA and *hhyL* genes was done by qPCR following Khdhiri et al. (2015).

### Quality control of sequencing reads

Our sequencing analysis pipeline using QIIME 1.8.0 (Kuczynski et al., 2011) software was based on the Itagger pipeline (Tremblay et al., 2015). V4 region Illumina sequencing reads were first processed using the *split_library* function implemented in QIIME for samples demultiplexing and forward primer removal. The assembly of paired-end reads was performed using FLASH software version 1.2.10 (Magoc & Salzberg, 2011). The software DUK (Li, Copeland & Han, 2011), a Kmer-based sequence matching tool, was then used to remove common sequencing contaminants as well as PhiX sequences added as control. Afterwards, sequences were trimmed to remove low quality ends and a general quality control was also applied. All reads containing at least a single *N* (ambiguous base), an average quality score of less than 30 or more than 15 bases with a quality score under 20 were discarded. The minimum and maximum read length were respectively set to 200 and 300 bases for the assembled paired-reads. Reverse primers were also removed using the *truncate*_*reverse*_*primer* function implemented in QIIME. Raw sequences were deposited in the Sequence Read Archive of the National Center for Biotechnology Information under the Bioproject PRJNA295403.

### Clustering and taxonomic identification of sequencing reads

Processed 2,554,543 high quality reads were clustered into OTUs (Operational Taxonomic Units) using the function *pick*_*otus* implemented in QIIME along with the USEARCH (Edgar, 2010) clustering algorithm. Sequences dereplication was performed at 100% sequence identity and clusters were formed by denoising unique sequences at 99% identity. Singletons were removed to avoid diversity bias. Remaining clusters were filtered using UCHIME (Edgar et al., 2011) chimera filter in *de novo* mode, followed by a reference-based filter step against the Gold reference database. These 2 chimera scanning steps allow a better removal of chimeras by removing sequences flagged as chimeras by any of the two methods. Chimera-checked clusters were then clustered into OTUs at 97% identity threshold also using the function *pick*_*otus* with the USEARCH software. Taxonomic identification of OTUs was performed using the *assign*_*taxonomy* function along with the naïve Bayesian Ribosomal Database Project (RDP) classifier 2.7 (Wang et al., 2007). Taxonomy was assigned based on the Greengenes taxonomy with the 16S rRNA Greengenes reference database (DeSantis et al., 2006). A rarefaction step was applied to the OTU libraries to standardize all libraries, by random subsampling, to the lowest amount of sequences (74,316 reads) to avoid bias introduced due to unequal sequencing efforts of the samples. This rarefied OTU table comprising the frequency distribution of the OTUs in each sample was used in downstream statistical and co-occurrence network analyses.

### Statistical analyses

Statistical analyses were performed using the software R version 3.0.2 (R Development Core Team, 2008). Pairwise comparison of H_2_ uptake activities in soil microcosms incubated under aH_2_ and eH_2_ exposure treatments was tested using one-way analysis of variance (ANOVA) and *post hoc* Tukey test. Shapiro–Wilk normality test was applied to confirm normal distribution of data before the ANOVA. The package “nlme” was used to compute nonlinear regression for modeling the first-order decay of *hhyL* gene abundance determined by qPCR. Discrimination of the samples according to their ribotyping profile was performed with the package “vegan.” Hellinger transformation of the OTU table (97% identity threshold) was computed due to the presence of zeros in the OTU table (Legendre & Gallagher, 2001). Euclidean distance matrix was used to generate an UPGMA agglomerative clustering of the samples. Identification of statistically different clusters was done by performing 999 permutations of the OTU table dataset separately across the samples and comparing the observed similarity score of each cluster against the expected values under the null hypothesis using the similarity profile tool (SIMPROF) implemented in the package “clustsig” 1.1 (Clarke, Somerfield & Gorley, 2008). Pairwise comparison of relative abundance of OTUs having a higher contribution than average to explain the two dimensions of the PCA space in soil microcosms incubated under aH_2_ and eH_2_ exposure treatments was tested using Kruskal–Wallis and *post hoc* Tukey test. Pairwise comparison of relative abundance of all OTUs in soil microcosms incubated under aH_2_ and eH_2_ exposure treatments was tested using the likelihood ratio test using the package “edgeR.” Covariation among OTUs during the incubation under controlled H_2_ levels was analyzed by correlation networks using the package “WGCNA” 1.41 (Langfelder & Horvath, 2008). A detailed methodology for the computation of correlation networks is provided in Text S1.

**Figure 1:**
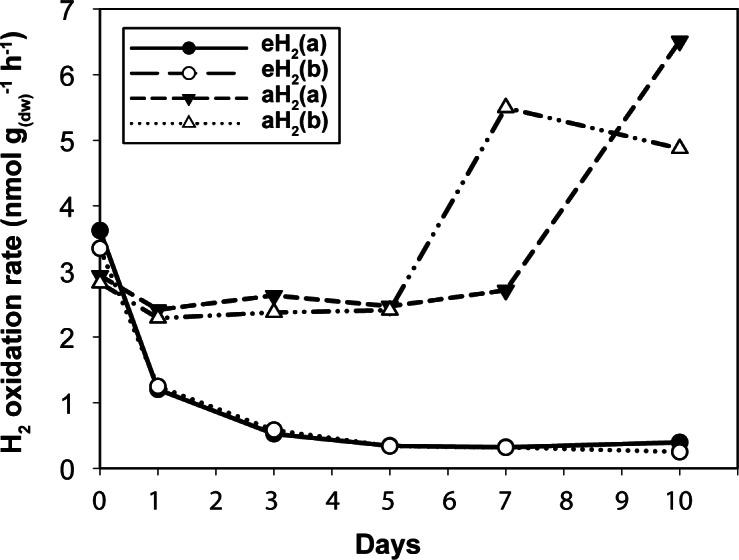
High affinity H_2_ oxidation rates. Time series of the high-affinity H_2_ oxidation rate measured in soil microcosms exposed to aH_2_ or eH_2_ throughout the incubation period.

**Figure 2:**
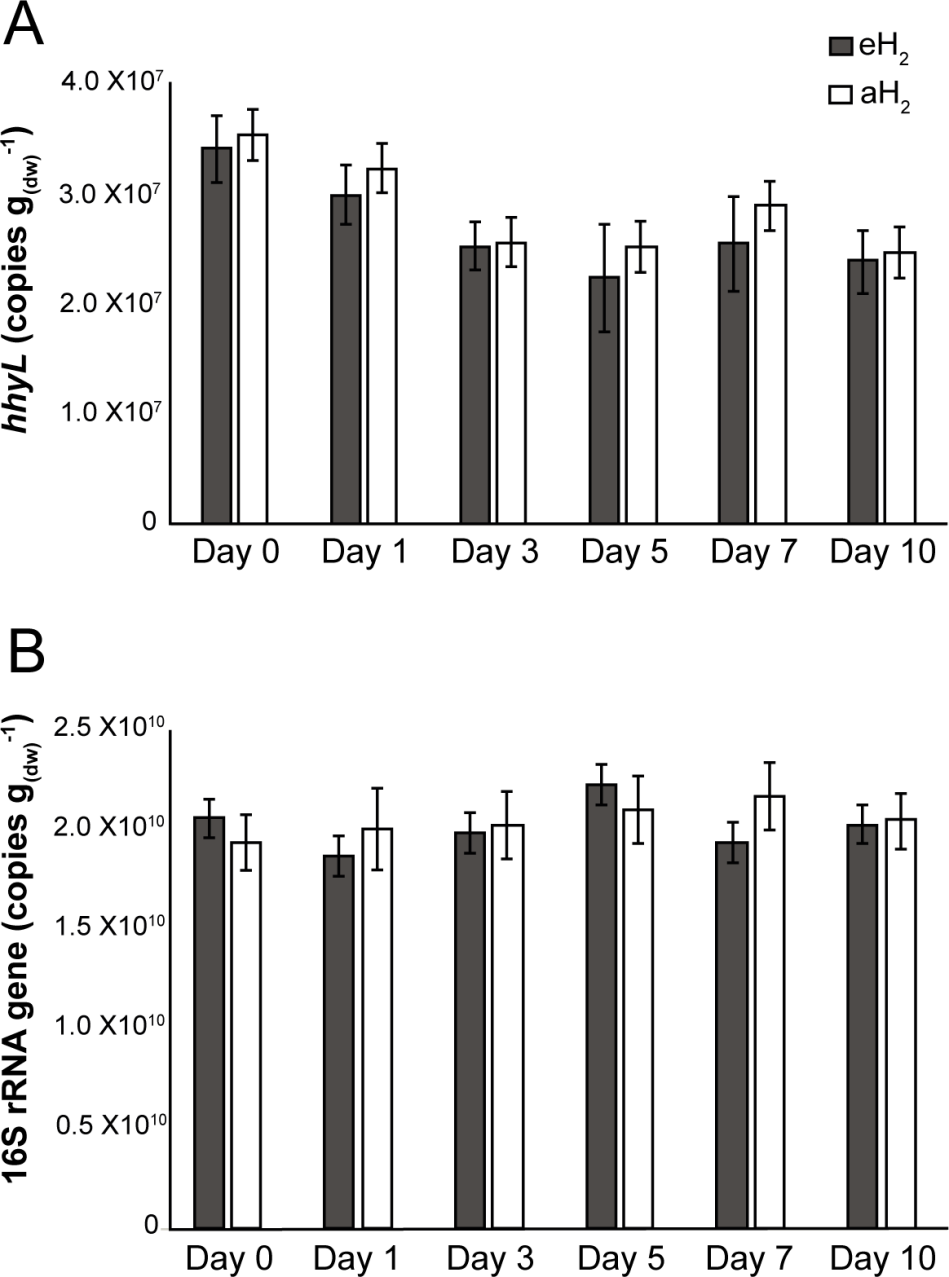
Time series of qPCR data. Time series of (A) *hhyL* and (B) 16S rRNA gene abundance in soil as determined by qPCR.

## Results and Discussion

### Impact of H_2_ exposure on the distribution and activity of HOB

Agricultural soil microcosms were incubated under two different H_2_ level treatments. The first treatment simulated soil exposure to atmospheric level of H_2_ (aH_2_; 0.54 ppmv), while the second simulated high affinity hydrogenases substrate saturation (eH_2_; 525 ppmv). High affinity H_2_ oxidation rates were not significantly different (ANOVA, *P* > 0.05) between the two pairs of microcosms at the beginning of the incubation (Fig. 1). The influence of H_2_ exposure could already be observed after 24 h. From hour 24 to day 10, a decline of high affinity H_2_ uptake rate was observed in microcosms exposed to eH_2_, while an increase of this uptake rate was measured in microcosms exposed to aH_2_ levels. This trend was maintained over the course of the incubation, with an oxidation rate of 6.2 ± 1.9 and $0.3\pm 0.1\hspace*{1em}\mathrm{nmol}\hspace*{1em}{\mathrm{g}}_{(\mathrm{dw})}^{-1}\hspace*{1em}{\mathrm{h}}^{-1}$ in aH_2_ and eH_2_ treatments at the end of the experiment, respectively. This response of H_2_ oxidation rate was accompanied by an alteration in the abundance of presumptive high affinity HOB in soil. The abundance of *hhyL* gene determined by qPCR did not change over the course of the incubation under aH_2_ (exponential regression, *P* = 0.06), while a slight but significant exponential decay (exponential regression, *P* = 0.02) was observed under eH_2_ (Fig. 2A). Indeed, the physiological role of high affinity hydrogenases differs within various taxonomic groups of bacteria. In streptomycetes, the enzyme is primarily expressed in spores to support a seed bank under a mixotrophic survival energy mode (Constant et al., 2010; Constant, Poissant & Villemur, 2008; Liot & Constant, 2015), while *Mycobacterium* express the enzyme in the exponential and stationary phases for mixotrophic growth as well as survival (Berney & Cook, 2010; Greening et al., 2015b; Greening et al., 2014b). Under axenic cultivation conditions, eH_2_ level would be expected to favour an increase of *Mycobacterium* biomass and persistence of streptomycetes spores. It should be noted that our qPCR data cannot differentiate between active and inactive cells but the decreasing trend of *hhyL* copy number in soil exposed to eH_2_ suggests that H_2_ was not sufficient to promote growth and persistence of high affinity HOB.

**Figure 3:**
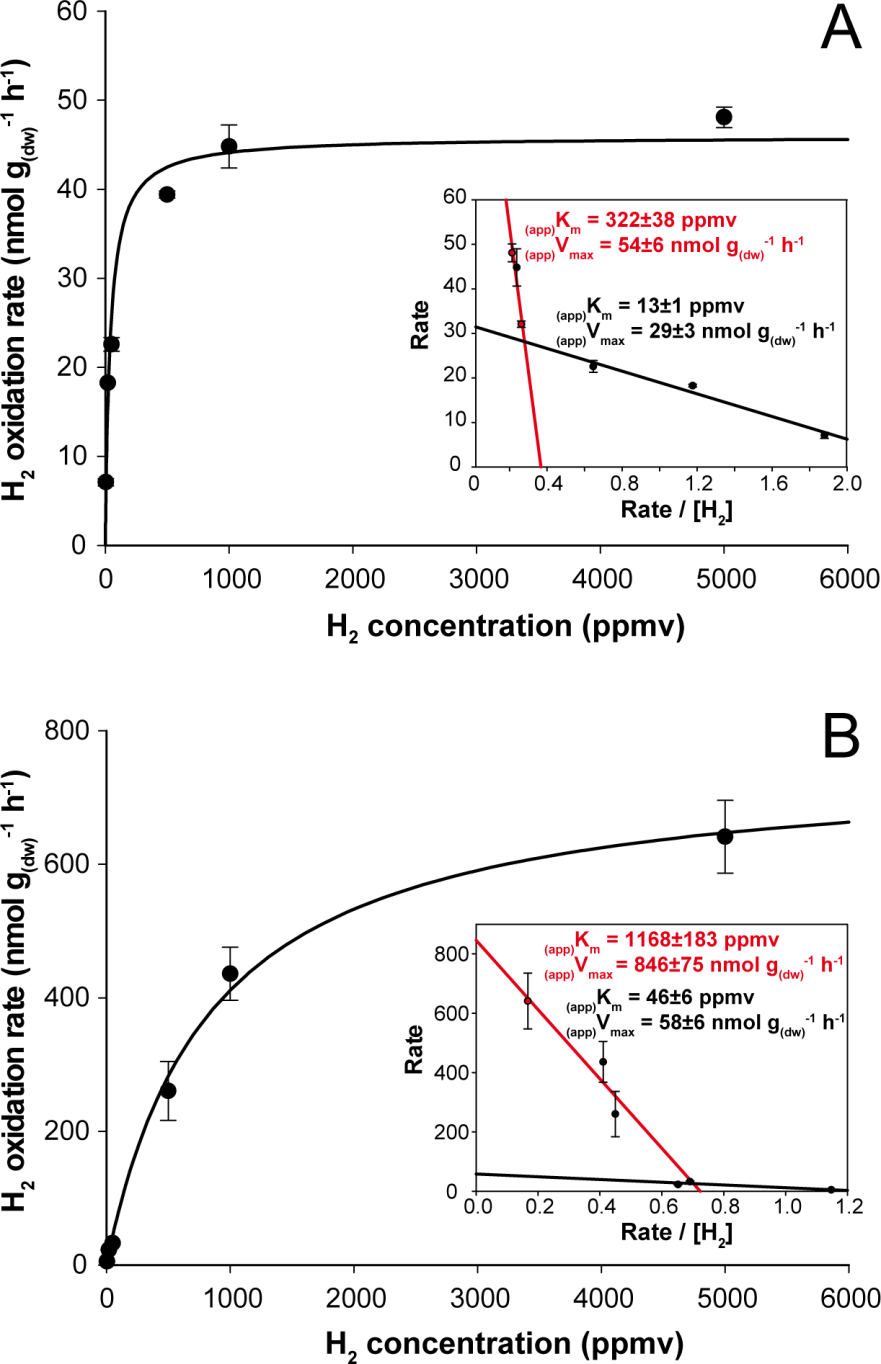
Kinetic parameters governing H_2_ oxidation activity in soil microcosms incubated under (A) aH_2_ and (B) eH_2_ exposure. Michaelis-Menten graphs are presented with Eadie-Hofstee plots highlighting the biphasic kinetic of the reaction in the inserts.

H_2_ soil exposure exerted a significant impact on the kinetic parameters governing H_2_ oxidation activity in soil. At the end of the incubation, microcosms exposed to aH_2_ level displayed _(app)_*K*_*m*_ of 40 ± 5 ppmv (Fig. 3A), while microcosms exposed to eH_2_ showed lower affinity towards H_2_ with an _(app)_*K*_*m*_ of 838 ± 163 ppmv (Fig. 3B). Similar observations were obtained by Dong & Layzell (2001), where soil exposed to elevated levels of H_2_ (600 ppmv) displayed an _(app)_*K*_*m*_ of 1,028 ppmv H_2_, while soil exposed to low H_2_ level (0.55 ppmv) was characterized by an _(app)_*K*_*m*_ of 40 ppmv. The biphasic kinetic parameters characteristics of low and high affinity HOB were also computed using Eadie-Hofstee plots to highlight high- and low-affinity H_2_ uptake activities in both H_2_ treatments. The H_2_ oxidation activity was mainly catalyzed by bacteria demonstrating intermediate and high-affinity in aH_2_ treatment (Fig. 3A, insert), while H_2_-oxidation activity by bacteria displaying low- and high-affinity for H_2_ were detectable in the eH_2_ treatment (Fig. 3B, insert). Low H_2_ exposure supported the metabolism of intermediate- to high-affinity HOB, such as some *Actinobacteria* species of *Streptomyces*, *Rhodococcus* and *Mycobacterium* (Berney et al., 2014; Constant et al., 2011b; Golding et al., 2012; Liot & Constant, 2015; Meredith et al., 2014; Schäfer, Friedrich & Lenza, 2013). Triggering of low-affinity H_2_ oxidation activity under eH_2_ treatment might be explained by knallgas bacteria such as *Proteobacteria* species encompassing *Ralstonia*, *Variovorax* and *Bradyrhizobium* using H_2_ for mixotrophic growth (Rittenberg & Goodman, 1969), as they are known to exhibit a H_2_ uptake threshold ranging from 1 to 200 ppmv H_2_ (Conrad, 1996).

### Impact of H_2_ exposure on soil bacterial community structure

**Figure 4:**
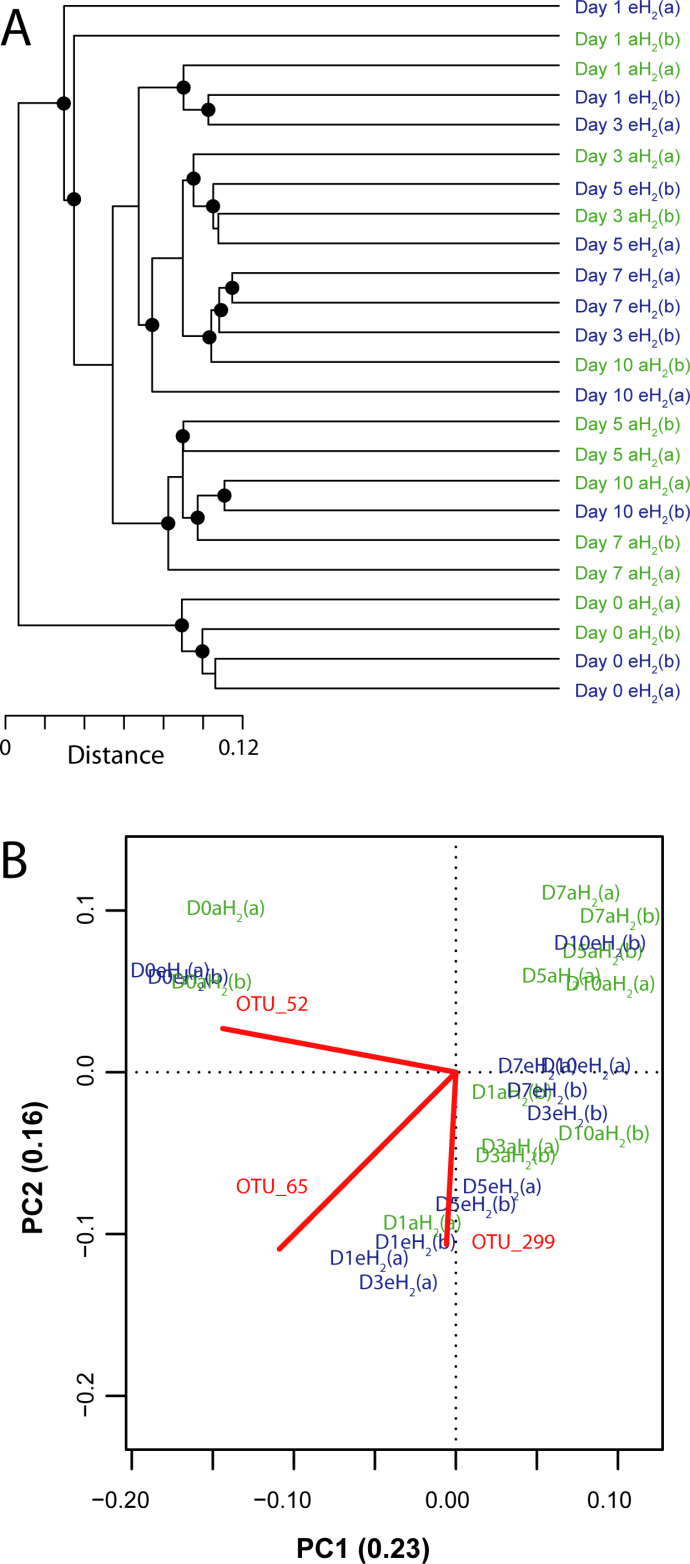
Influence of H_2_ exposure on bacterial ribotyping profile. (A) UPGMA agglomerative clustering of soil samples derived from a matrix of Euclidean distance calculated after Hellinger transformation of OTU (97% identity) absolute abundance in soil microcosms exposed to eH_2_ and aH_2_ throughout a 10-day incubation period. The black circles ● represent significant nodes (*P* ≤ 0.05). (B) Principal component analysis showing the distribution of soil subsamples in a reduced space defined by the relative abundance of 16S rRNA gene sequences classified at the OTU level (97% identity). Only the OTUs (represented by red lines) having a higher contribution than average to explain the two dimensions of the PCA space are shown to facilitate visualization of the analysis. Soil subsamples collected in microcosms exposed to eH_2_ and aH_2_ are shown in blue and green, respectively.

Soil subsamples were collected in the microcosms during the incubation period to evaluate the temporal variation of soil bacterial community structure. H_2_ treatment did not influence the abundance of 16S rRNA gene in soil (Fig. 2B). This is in contrast with another study that reported more than twofold increase of the amount of bacterial cells upon elevated H_2_ exposure, but a mistake in the reported H_2_ concentration units by the authors (i.e., the use of a gas mixture of 2,000 ml H_2_ per L of synthetic air) impairs a sound comparison with the present investigation (Stein et al., 2005). High-throughput sequencing of PCR-amplified 16S rRNA gene unveiled that bacterial communities were dominated by *Proteobacteria* (34%), *Acidobacteria* (20%), *Actinobacteria* (10%), *Verrucomicrobia* (8%) and *Bacteroidetes* (5%) (Fig. S2). Species richness was not affected by H_2_ exposure (ANOVA, *P* > 0.05), with Shannon indices of 9.38 ± 0.05 and 9.34 ± 0.17 for aH_2_ and eH_2_ treatment respectively. An UPGMA hierarchical clustering analysis was computed to compare bacterial community profiles from soil subsamples collected in microcosms exposed to different H_2_ concentrations. The four microcosms encompassed the same cluster before the incubation, indicating high similarity of their initial ribotyping profile (Fig. 4A). No coherent impact of H_2_ exposure on microbial community structure was observed after 1 and 3 incubation days. Ribotyping profiles obtained for sub-samples collected after 5 and 7 incubation days were separated with confidence in different clusters, suggesting a response of microbial communities to H_2_ exposure. This discrimination was however transient as ribotyping profiles corresponding to the last incubation day could not be discriminated according to their respective H_2_ treatments (Fig. 4A). The transient response was likely due to the fact that H_2_ alone was not sufficient to maintain a sustained change in microbial community structure. Decrease of complex carbon sources and nutrients used along with H_2_ for mixotrophic growth in soil is a potential explanation for the convergence of the ribotyping profiles after 7 incubations days.

**Figure 5:**
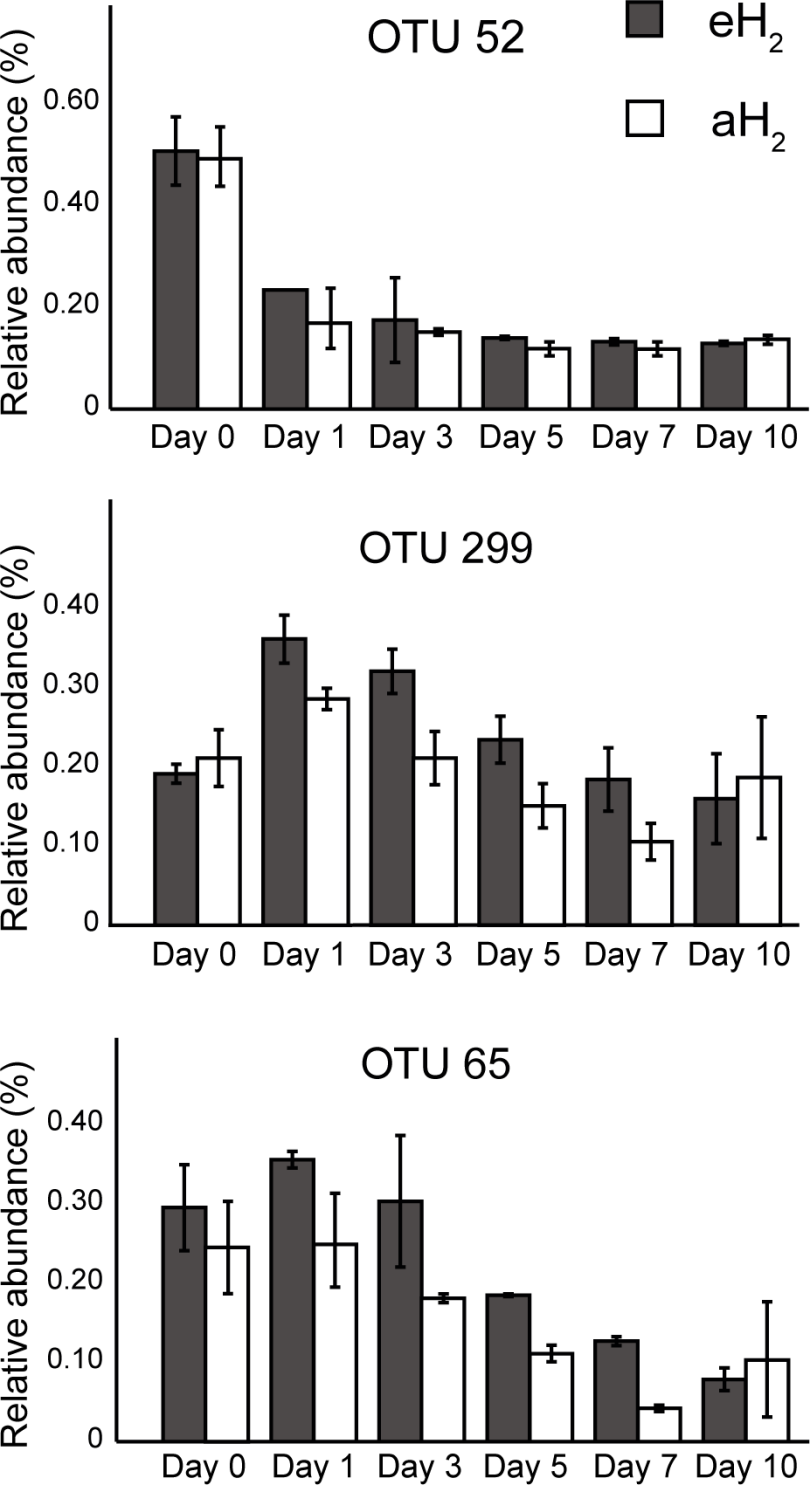
OTUs of interest to explain the PCA. Time series of the relative abundance of the 3 OTUs having a higher contribution than average to explain the two dimensions of the PCA space in soil microcosms. The average and standard deviation measured in replicated microcosms are represented. The closest taxonomic affiliations of OTUs 52, 299 and 65 are, respectively, the bacterial order *MND1* (*Betaproteobacteria*), genus *Bacillus* (*Firmicutes*) and the species *Bacillus cereus* (*Firmicutes*).

A PCA was computed to identify OTUs contributing to the clusterization of ribotyping profiles. The ordination space defined by the first two components explained 38.6% of the variation observed (Fig. 4B). Three OTUs defined an important proportion of the reduced space represented by both axes (Fig. 5). The relative abundance of OTU 52 (classified as a member of the class *Betaproteobacteria*) contributed to distinguish the four soil subsamples collected before the incubation from the 20 other subsamples collected after 1, 3, 5, 7 and 10 incubation days along the first axis as it decreased after the beginning of the incubation period (Kruskal–Wallis, *P* < 0.05). Two OTUs classified as members of the order *Bacillales* (OTUs 65 and 299) contributed to discriminate both H_2_ treatments in the ordination space after 5 and 7 incubation days (Kruskal–Wallis, *P* < 0.05). These OTUs were more abundant in microcosms exposed to eH_2_ until day 10, where they reached a similar relative abundance in aH_2_ and eH_2_ treatments. The transient response of these OTUs in eH_2_ treatment could be a direct consequence of H_2_ exposure as representatives of the genus *Bacillus* possess putative membrane-bound type 1a and 1d [NiFe]-hydrogenases (Greening et al., 2016).

Conflicting results were obtained in two different studies reporting the impact of H_2_ soil exposure on bacterial community structure based on 16S rRNA terminal restriction fragment profiles analysis. H_2_ exerted no incidence on ribotyping profile in soil exposed to 250 nmol H_2_cm^−3^h^−1^ (500 ppmv H_2_ in artificial air, added at 45 ml min^−1^), while a single ribotype related to *Mycobacterium* increased in soils upon elevated H_2_ exposure (Osborne, Peoples & Janssen, 2010). On the other hand, an exposure rate of 33 nmol cm^−3^ h^−1^ (79 ppmv H_2_ at 100 cm^3^ min^−1^) exerted a significant influence on bacterial community profile, with an increase of T-RFLP peaks belonging to *γ*-*Proteobacteria* and a decrease of peaks belonging to *Actinobacteria* and *α*-*Proteobacteria* upon elevated H_2_ exposure (Zhang, He & Dong, 2009). A likelihood ratio analysis, fitting a negative binomial generalized log-linear model to the sequencing data (McCarthy, Chen & Smyth, 2012; Robinson, McCarthy & Smyth, 2010), unveiled that distribution of 958 OTUs was influenced by H_2_ exposure. The relative abundance of OTUs responding to H_2_ treatment ranged between 0.001 and 1.8%, suggesting an incidence of H_2_ on members of the rare biosphere and abundant taxa. The influence of H_2_ was uneven among different taxonomic groups as different representatives of the same taxa (i.e., OTUs classified at the phylum and the order taxonomic levels) were found to be favored or disfavored in response to eH_2_ exposure (Table 1). Even though the uneven response among taxonomic groups impairs prediction of H_2_ exposure on metabolic functions in soil (Langille et al., 2013), this observation is sufficient to demonstrate that previous investigations relying on low-resolution community fingerprinting techniques have considerably underestimated the response of soil microbial communities to H_2_ exposure.

**Table 1:** Summary of OTUs showing different relative abundance in eH_2_ and aH_2_ treatments (Likelihood ratio test, *P* < 0.05). Altogether, 406 OTUs were more abundant in eH_2_ and 552 were more abundant in aH_2_. The eH_2_ and aH_2_ rows indicate the treatment in which the identified phylotypes are more abundant. A single or the two most abundant OTUs are identified for each phylum. A list of the 958 OTUs whose distribution was influenced by H_2_ treatments is provided in the raw data file accompanying the article.

Treatments	Phyla	Most abundant OTU (order level)
eH_2_	*Proteobacteria* (27.6%)	*Myxococcales*
	*Planctomycetes* (10.6%)	*Gemmatales, Phycisphaerales*
	*Bacteroidetes* (9.6%)	*Sphingobacteriales*
	*Chloroflexi* (9.1%)	*Ktedonobacteria*
	*Acidobacteria* (7.1%)	*Acidobacteriales, Solibacterales*
	*Verrucomicrobia* (6.2%)	*Verrucomicrobiales*
	*Actinobacteria* (5.9%)	*Actinomycetales, Solirubrobacterales*
	*Elusimicrobia* (3.0%)	*FAC88*
	*Gemmatimonadetes* (2.7%)	*Gemmatimonadales*
	*Firmicutes* (1.5%)	*Clostridiales, Bacillales*
	*Archaea* (1.0%)	*Methanobacteriales*
	*Armatimonadetes* (0.7%)	*CH21*
	*Chlorobi* (0.2%)	*SM1B09*
	Others (14.8%)	–
aH_2_	*Proteobacteria* (33.9%)	*Rhodospirillales, Myxococcales*
	*Acidobacteria* (11.4%)	*Solibacterales*
	*Planctomycetes* (11.1%)	*Gemmatales*
	*Chloroflexi* (6.2%)	*Ktedonobacteria, A4b*
	*Bacteroidetes* (5.1%)	*Sphingobacteriales*
	*Actinobacteria* (4.3%)	*Actinomycetales, Acidimicrobiales*
	*Firmicutes* (3.8%)	*Clostridiales*
	*Verrucomicrobia* (3.4%)	*Spartobacteriales*
	*Gemmatimonadetes* (2.7%)	*Gemmatimonadales*
	*Archaea* (1.8%)	*SD-NA (Crenarchaeota)*
	*Elusimicrobia* (1.4%)	*Elusimicrobiales, MVP-88*
	*Armatimonadetes* (1.3%)	*Chthonomonadales, CH21*
	*Chlorobi* (0.9%)	*SM1B09*
	Others (12.7%)	–

### Impact of H_2_ exposure on the co-occurrence of OTUs

Correlation networks were computed to investigate the impact of H_2_ exposure on the covariation of OTUs throughout the incubation period and identify OTUs for which the distribution is influenced by H_2_. Two separate networks were computed: the first for elevated H_2_ exposure (eH_2_ network) and a second for low H_2_ exposure (aH_2_ network). Both networks contained 43 modules and module preservation statistics demonstrated that module composition significantly changed across H_2_ treatments (Text S1). The significances of measured H_2_ oxidation rate in explaining network structure were 0.33 and 0.98 in aH_2_ and eH_2_ networks, again pointing out a significant impact of H_2_ on soil microbial community structure. Indeed, module eigengenes of five modules were significantly correlated with high-affinity H_2_ oxidation rate time series in eH_2_ network, while no module was related to the activity in aH_2_ network (Fig. S3). Together, these 5 modules represent 1,140 OTUs, which is more than a third of the whole eH_2_ network (3,154 OTUs). OTUs belonging to one module were hindered by eH_2_ exposure, while members of the four other modules were favored by eH_2_ treatment. The OTUs belonging to these five modules were members of the rare biosphere as well as more abundant ribotypes, with relative abundance ranging between 0.001% and 4.3% encompassing *Proteobacteria*, *Chloroflexi*, *Acidobacteria* as well as other phyla (Fig. S4). Clustering of these OTUs at the class and order levels unveiled that none of the taxonomic groups were restricted to the five modules correlated with H_2_ oxidation rate, supporting the previous observation that response to H_2_ exposure and the distribution of hydrogenase genes is uneven within each taxonomic group. The 38 modules in eH_2_ network whose eigengene showed no significant correlation with H_2_ oxidation rate are ecologically relevant observations since they represent indirect impacts of H_2_ exposure on soil microbial communities and highlight a previously overlooked role of H_2_ in shaping potential microbe-microbe cooperation and competition interactions. Deciphering the impact of these complex interactions on soil biogeochemical processes was beyond the scope of this study but will deserve attention in future investigations.

## Conclusion

In conclusion, this exploratory study validated our hypothesis that elevated H_2_ exposure influences the activity of HOB, leading to direct impacts on the HOB but also indirect impact on the whole soil bacterial community through competitive and synergistic microbe-microbe interactions. Indeed, H_2_ soil exposure has an impact on the ecological niche of bacteria unevenly distributed among taxonomic groups beyond the alteration of HOB reported in early investigations. Our study was limited to a single farmland soil, with two replicated microcosms per treatment. Nonetheless, considering the significant impact that H_2_ exposure has brought to soil bacterial communities, it is reasonable to expect a similar effect on other soil types. Considering the steep H_2_ concentration gradient surrounding N_2_-fixing nodules, from 20,000 ppmv to sub-atmospheric levels along a 4.5 cm radius, the response of microorganisms might vary as a function of the distance from H_2_ diffusing sources as well as soil types, physicochemical conditions and the structural and functional structure of microbial community (Hartmann et al., 2015). The impact of H_2_ and HOB on soil microbial communities will definitely deserve more attention in soil microbiology. There is a need to further study whether elevated H_2_ exposure also exerts noticeable changes in soil ecological functions other than H_2_ oxidation, such as carbon metabolism, nutrient cycling, trace gas exchanges and xenobiotic degradation. Such a significant impact of a gas molecule on soil was observed in a study investigating the impact of a doubling atmospheric CO_2_ concentration, simulating climate change, on microbial communities (Zhou et al., 2011). A metagenomics approach on the matter might provide further answers on this rather ubiquitous microniche as well as the succession of hydrogenases along H_2_ concentration gradients in the environment.

## Supplemental Information

10.7717/peerj.1782/supp-1 Supplemental Information 1 Raw data All raw data utilized for this manuscript are provided as supplementary material (H2 oxidation activities, soil physicochemical properties, list of OTUs whose distribution was affected by H2 treatments).

10.7717/peerj.1782/supp-2 Text S1 Supplementary methods for correlation networks Supplementary information for the computation of correlation networks.

10.7717/peerj.1782/supp-3 Figure S1 Schematic representation of the dynamic microcosm chambers (A) Schematic representation of the dynamic microcosm chambers utilized in this study to expose soil to aH_2_ or eH_2_ levels. (B) Photograph of one soil microcosm.

10.7717/peerj.1782/supp-4 Figure S2 Taxonomic profiles in soil Taxonomic profiles of the OTUs clustered at the phylum level for each microcosm at the beginning (day 0) and at the end of the incubation (day 10).

10.7717/peerj.1782/supp-5 Figure S3 Module-Trait relationship heatmap for correlation network computed using OTU covariation profile under eH_2_ or aH_2_ exposure Module-Trait relationship heatmap for correlation network computed using OTU covariation profile under eH_2_ or aH_2_ exposure. Each line in the heatmaps corresponds to a module. The colors in the heatmap stand for the Spearman correlation coefficient between the module eigengene and high affinity H_2_-oxidation rate measured in the microcosms. One module out of the 5 has the opposite sign (M1; red color in the heatmap eH_2_) because it is the only one showing a positive correlation (*P* < 0.05) with high affinity H_2_-oxidation rate. In contrast, the others modules (M2–M5; green color in the heatmap eH_2_) displayed a negative correlation (*P* < 0.05) with high affinity H_2_-oxidation rate.

10.7717/peerj.1782/supp-6 Figure S4 Taxonomic profile of OTUs found in selected modules of correlation networks Distribution of the relative abundance of the most abundant phyla in modules whose eigengene is significantly correlated with H_2_ oxidation rate in the eH_2_ network.

10.7717/peerj.1782/supp-7 Table S1 Primers for sequencing bacterial 16S rRNA gene List of the barcodes used to prepare bacterial 16S rRNA gene PCR amplicon libraries.

## Additional Information and Declarations

### DNA Deposition

The following information was supplied regarding the deposition of DNA sequences: Raw sequences were deposited in the Sequence Read Archive of the National Center for Biotechnology Information under the Bioproject PRJNA295403.

### Data Availability

The following information was supplied regarding data availability: Raw data an be found in the Supplemental Information.
