# Supplementary material for: H2-saturation of high affinity H2-oxidizing bacteria alters the ecological niche of soil microorganisms unevenly among taxonomic groups
Source: PeerJ. 2016 Mar 10;4:e1782. doi: 10.7717/peerj.1782 (PMC4793312; doi:10.7717/peerj.1782)
Supplement: Text S1 — Supplementary information for the computation of correlation networks. [file peerj-04-1782-s002.docx]

# Text S1. Supplementary information for the computation of correlation networks

A) Construction of weighted co-occurrence networks

Covariation among OTUs during the incubation under controlled H_2_ levels was analyzed by correlation networks using the package “WGCNA” 1.41 (Langfelder & Horvath 2008). After sequencing, the output OTU table was divided in two distinct datasets: the eH_2_ exposure treatment (n = 12) and the aH_2_ exposure treatment (n = 12). Both datasets were analyzed separately, resulting in the computation of two independent weighted co-occurrence networks. A pairwise Spearman correlation matrix (similarity matrix) was calculated between all OTUs. An adjacency matrix was then calculated by raising the correlation coefficient of the similarity matrix to the soft-thresholding power 12 (ß = 12, eH_2_ treatment) or 14 (ß = 14, aH_2_ treatment), as detailed in Horvath *et al* (Langfelder & Horvath 2008; Langfelder et al. 2011). This transformation resulted in a distribution of OTU connectivity conforming to the scale-free topological model - linear regression model fitting of log_10_-frequency distribution of the connectivity as a function of log_10_-connectivities resulted in slopes of -1 and R^2^ above 0.80 (Zhang & Horvath 2005). The adjacency matrix was used to delineate modules including OTUs showing highly similar distribution profile throughout the incubation, with a minimum size threshold of 10 OTUs per module. Module eigengene was computed for each module and those showing dissimilarities lower than 0.40 were merged together. The consensus modules alignment method (Langfelder et al. 2011) implemented in WGCNA was used to insure that both replicates of each treatment produced similar networks (*i.e.* modular patterns were conserved across replicates). This technique uses the cross-tabulation preservation statistics described in the next section.

## *B) Comparison of networks patterns across treatments*

Module preservation statistics were used to identify preserved modules among networks. Two methods were used in order to assess modules preservation between aH_2_ and eH_2_ networks: cross-tabulation based and composite based preservation statistics. These methods allowed to statistically compare the structure of both networks and assert with confidence whether H_2_ had an impact on the structure of the soil bacterial community. The first method, cross-tabulation based statistics, requires module detection of the reference (aH_2_) and test (eH_2_) networks as input for the comparison of modules assignments between both networks. The bias of this analysis is that the module separation has already been done in both networks and that the pairwise comparisons are limited. The overlap of nodes between each pair of modules from the aH_2_ and eH_2_ networks is then calculated. A contingency table (heatmap) representing these overlaps (data not shown) shows which modules are similar between both networks. Significant module preservation was considered when P ≤ 0.05. In this case, 4 out of 43 modules were considered similar between both treatments. None of those modules was characterized by eigengene value displaying significant correlation with H_2_ oxidation rate.

The second method used to identify preserved modules among aH_2_ and eH_2_ networks was composite preservation statistics. This analysis summarizes 4 density (Z_density_) and 3 connectivity (Z_connectivity_) based preservation statistics into a single variable, Z_summary_ (Langfelder et al. 2011). Z_density_ measures if densely connected nodes in the control network remain as densely connected in the test (eH_2_) network, while Z_connectivity_ measures if connectivity patterns are similar in both networks. It only requires the module assignment of the reference data (control network) and the adjacency matrix from the test data. This method reduces the bias of cross-tabulation based statistics since it does not need to compare a limited set of modules in the test network. In our analysis, we used the Z_summary_ and medianRank variables. 999 permutations were applied to these calculations. Thresholds defining module similarity between the two networks were: Z_summary_ > 10 for highly preserved modules, Z_summary_ between 2 and 10 for weakly to moderately preserved modules and Z_summary_ < 2 for modules that are not preserved between networks (Langfelder et al. 2011). In our study, a single module was strongly preserved while 6 were weakly to moderately preserved (Figure Text S1A). The medianRank preservation statistic was also used to complement Z_summary_, as it is less dependent on module size than Z_summary_. Modules with a median rank closer to zero are more preserved than those with higher values. This statistic identified the same preserved modules as the Z_summary_ approach (Figure Text S1A).

*C) Conluding remarks regarding the impact of H_2_ exposure on OTU co-occurrence – Only a few modules are conserved between aH_2_ and eH_2_ networks*

By complementing cross-tabulation based preservation statistics with composite (comprising density and connectivity based statistics) based statistics, we have insured that we could detect modules that have a high proportion of similar nodes, that are as densely connected and that show similar connectivity patterns in both networks. Only 4 modules were considered similar (Fisher’s exact test, α = 0.05) between both networks when using cross-tabulation based statistics while 1 module was considered highly preserved (Z_summary_ ≥ 10) and 6 were weakly to moderately preserved (2 < Z_summary_ < 10) according to composite based preservation statistics. All modules identified with the first method were identified using the composite based preservation statistics as well. A high proportion of the modules were considered unique (36 out of 43) which suggests that the whole network structure has been altered by H_2_. On the other hand, cross-tabulation module preservation analysis showed that replicated microcosms shared the same modules, hence they were reproducible replicates.

**Figure Text S1**. Composite preservation statistics of modules from the eH_2_ network (test network) against modules from the aH_2_ network (reference network). Dots represent the various modules in the reference network. (a) Z_summary_ values of each module as a function of module size (number of nodes). The blue and green dash lines represent minima and maxima of the two thresholds indicating module preservation. Z_summary_ > 10 indicates a strong preservation, while 2 ≤ Z_summary_ ≤ 10 indicates a weak to moderate module preservation between networks. Z_summary_ < 2 indicates unpreserved modules. (b) Median rank values of each module as a function of module size (number of nodes). Lower Median ranks (close to 0) indicate higher module preservation.


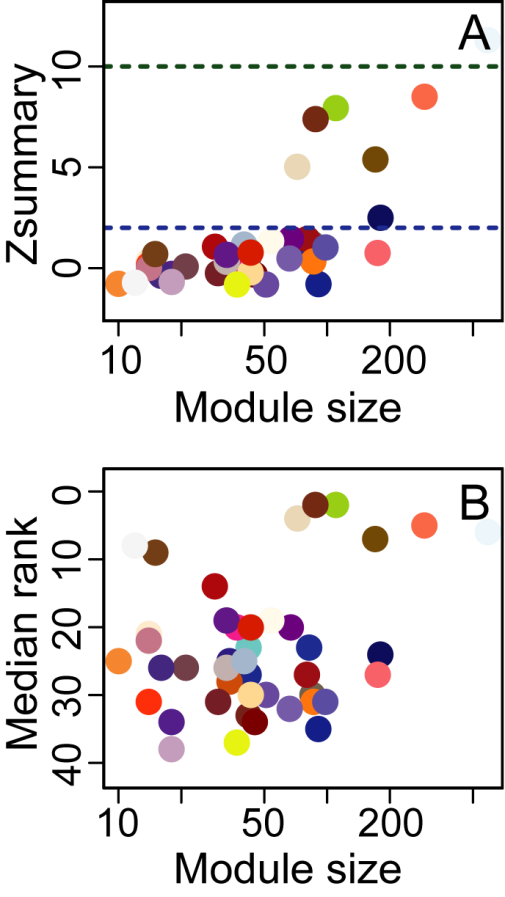


**References**

Langfelder P, and Horvath S. 2008. WGCNA: an R package for weighted correlation network analysis. *BMC Bioinformatics* 9:559.

Langfelder P, Luo R, Oldham MC, and Horvath S. 2011. Is my network module preserved and reproducible? *PLoS Computational Biology* 7:e1001057. 10.1371/journal.pcbi.1001057

Zhang B, and Horvath S. 2005. A general framework for weighted gene co-expression network analysis. *Statistical applications in genetics and molecular biology* 4.
