# Supplementary figures and images for: H2-saturation of high affinity H2-oxidizing bacteria alters the ecological niche of soil microorganisms unevenly among taxonomic groups
Source: PeerJ. 2016 Mar 10;4:e1782. doi: 10.7717/peerj.1782 (PMC4793312; doi:10.7717/peerj.1782)

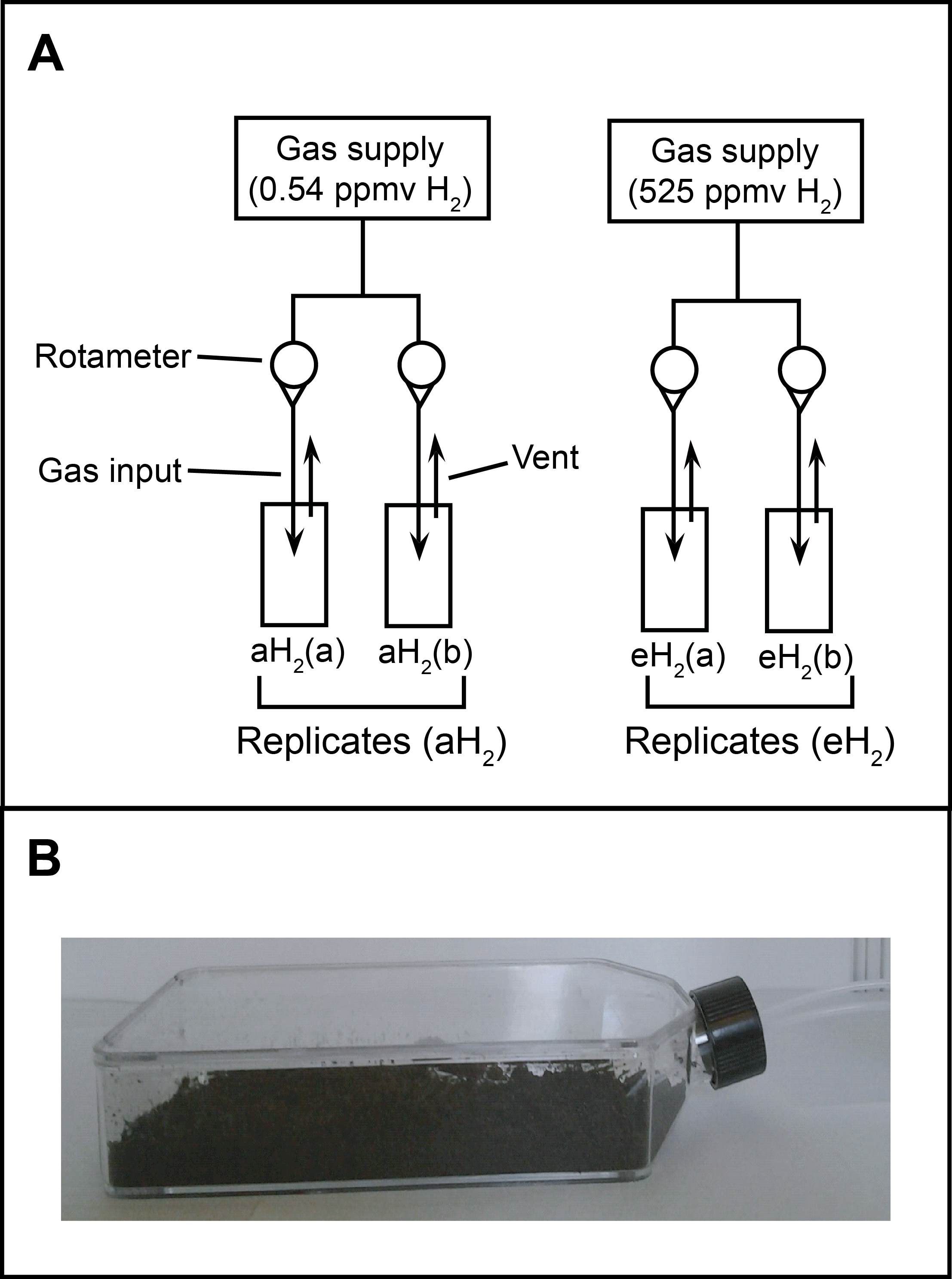

Supplement: Figure S1 — (A) Schematic representation of the dynamic microcosm chambers utilized in this study to expose soil to aH2 or eH2 levels. (B) Photograph of one soil microcosm. [file peerj-04-1782-s003.png]

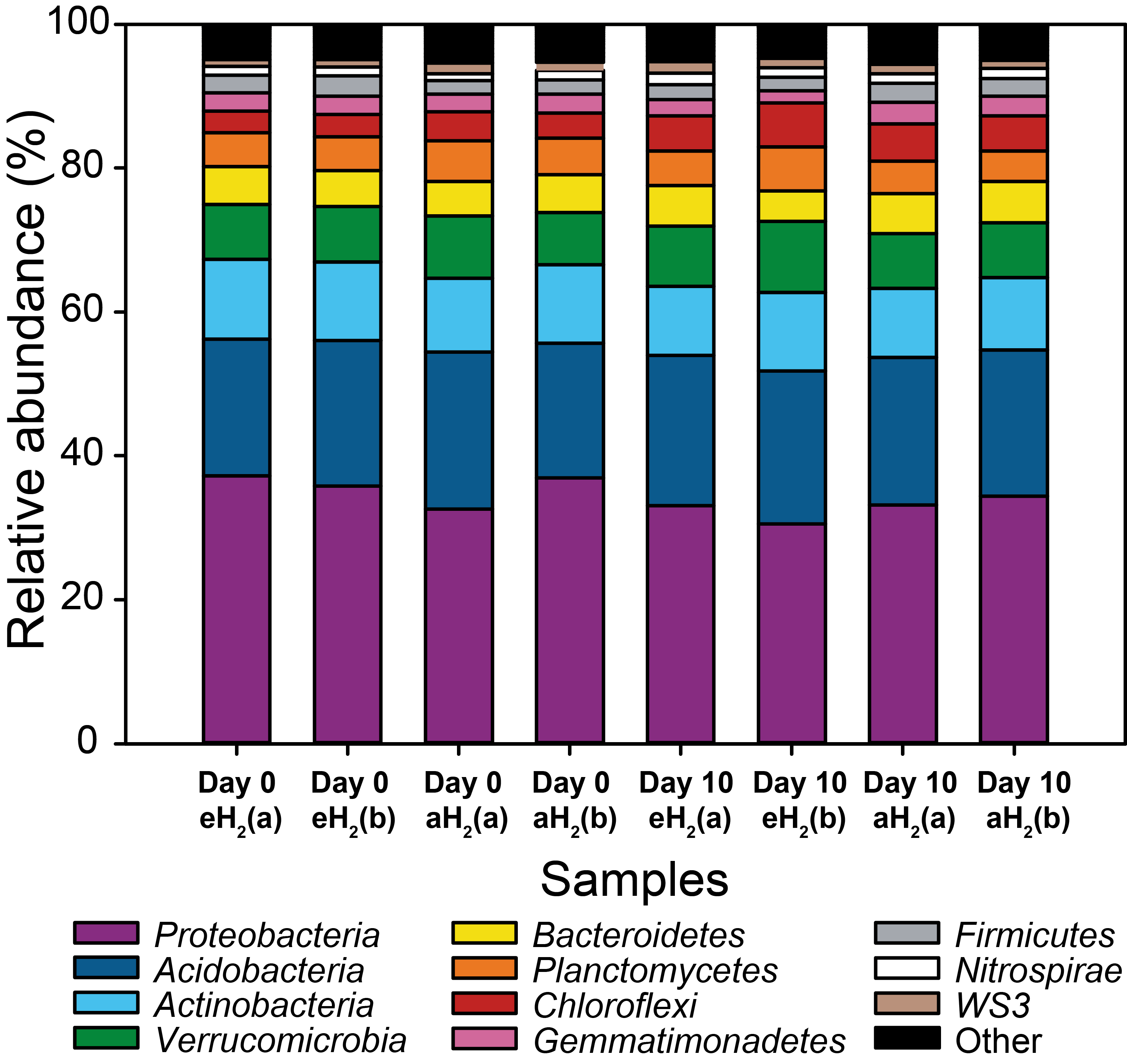

Supplement: Figure S2 — Taxonomic profiles of the OTUs clustered at the phylum level for each microcosm at the beginning (day 0) and at the end of the incubation (day 10). [file peerj-04-1782-s004.png]

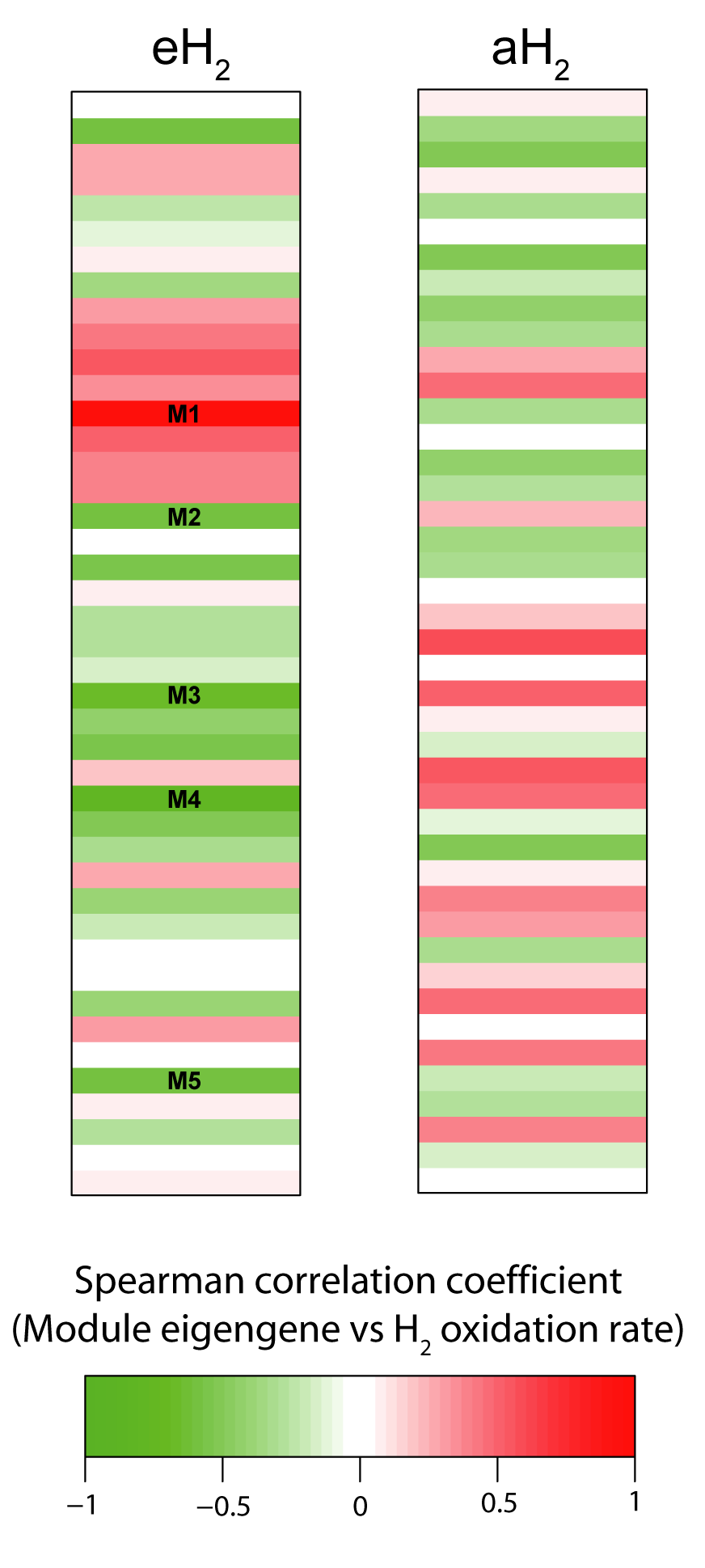

Supplement: Figure S3 — Module-Trait relationship heatmap for correlation network computed using OTU covariation profile under eH2 or aH2 exposure. Each line in the heatmaps corresponds to a module. The colors in the heatmap stand for the Spearman correlation coefficient between the module eigengene and high affinity H2-oxidation rate measured in the microcosms. One module out of the 5 has the opposite sign (M1; red color in the heatmap eH2) because it is the only one showing a positive correlation (P < 0.05) with high affinity H2-oxidation rate. In contrast, the others modules (M2–M5; green color in the heatmap eH2) displayed a negative correlation (P < 0.05) with high affinity H2-oxidation rate. [file peerj-04-1782-s005.png]

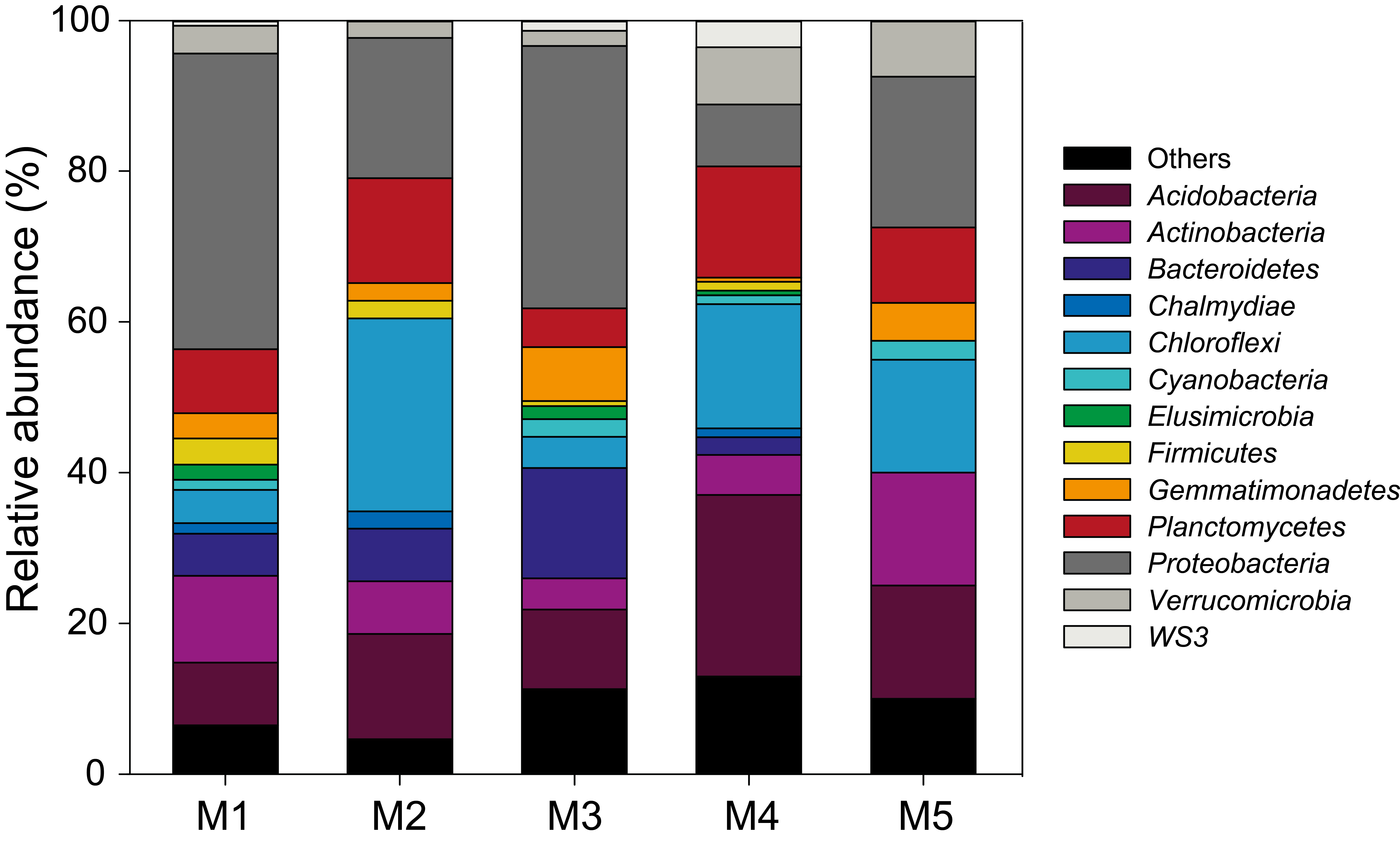

Supplement: Figure S4 — Distribution of the relative abundance of the most abundant phyla in modules whose eigengene is significantly correlated with H2 oxidation rate in the eH2 network. [file peerj-04-1782-s006.png]
