# Supplementary material for: H2-saturation of high affinity H2-oxidizing bacteria alters the ecological niche of soil microorganisms unevenly among taxonomic groups
Source: PeerJ. 2016 Mar 10;4:e1782. doi: 10.7717/peerj.1782 (PMC4793312; doi:10.7717/peerj.1782)
Supplement: Table S1 — List of the barcodes used to prepare bacterial 16S rRNA gene PCR amplicon libraries. [file peerj-04-1782-s007.docx]

Table S1. List of the barcodes used to prepare bacterial 16S rRNA gene PCR amplicon libraries^A^.

| Library  (internal ID) | Library  (sample name) | Barcode |
| --- | --- | --- |
| D1SA | D0-eH_2_(a) | GCATGGCTCTA |
| D1SB | D0-eH_2_(b) | AGGCGACCTTA |
| D1SC | D0-aH_2_(a) | GATAGTGCCAC |
| D1SD | D0- aH_2_ (b) | GAACACTTCTG |
| D2SA | D1-eH_2_(a) | TACCGCTAGTA |
| D2SB | D1-eH_2_(b) | TCTGGAACGCT |
| D2SC | D1- aH_2_ (a) | GGTATGACTCA |
| D2SD | D1- aH_2_ (b) | CTTGTAGGACC |
| D3SA | D3-eH_2_(a) | ACTGTACGCGT |
| D3SB | D3-eH_2_(b) | TACAGATGGCT |
| D3SC | D3- aH_2_ (a) | TGCGAACGTAT |
| D3SD | D3- aH_2_ (b) | TTGAACCAGCT |
| D4SA | D5-eH_2_(a) | TCAGGCGCTTA |
| D4SB | D5-eH_2_(b) | ATACTTCGCAG |
| D4SC | D5- aH_2_ (a) | TGTCGGCTACA |
| D4SD | D5- aH_2_ (b) | TGGCTCTACAG |
| D5SA | D7-eH_2_(a) | GCCGCTTAATA |
| D5SB | D7-eH_2_(b) | GTTACCTCAGA |
| D5SC | D7- aH_2_ (a) | GTACGAATCCT |
| D5SD | D7- aH_2_ (b) | TCCTAGCAGTG |
| D6SA | D10-eH_2_(a) | CACGTGACATG |
| D6SB | D10-eH_2_(b) | CTTCGGCAGAA |
| D6SC | D10-Air(a) | CATCAGCGTGT |
| D6SD | D10-Air(b) | CCAGTGTATGC |

^A^The V4 region of 16S rRNA gene was amplified using the primers:

- 515F (5’-GTGCCAGCMGCCGCGGTAA-3) and;
- 805R (5’-GGACTACHVGGGTWTCTAAT-3’).
